# Honeybee aging as an emergent property of a self-organizing dissipative system

**DOI:** 10.3389/fsysb.2026.1874982

**Published:** 2026-07-20

**Authors:** Radmila Čapková Frydrychová

**Affiliations:** 1 Faculty of Agriculture and Technology, University of South Bohemia, České Budějovice, Czechia; 2 Institute of Entomology, Biology Centre, Czech Academy of Sciences, České Budějovice, Czechia

**Keywords:** aging, dissipative structure, honeybees, juvenile hormone, superorganism, vitellogenin

## Abstract

The honeybee colony provides a remarkable example of aging plasticity emerging from collective biological organization. Through the reciprocal regulation of juvenile hormone (JH) and vitellogenin (Vg), honeybees demonstrate that aging is a dynamic and reversible process shaped by social and environmental conditions. The JH–Vg axis acts as a biological metronome that coordinates behavioral state, lifespan, and seasonal colony dynamics, synchronizing colony persistence with ecological and planetary rhythms. At the colony level, physiological states are continuously adjusted through interactions among endocrine regulation, pheromonal communication, and environmental cues. As a result, the colony maintains its organization not through static equilibrium, but through ongoing transitions between alternative physiological states that support either rapid growth or long-term survival. In this sense, the honeybee superorganism can be viewed as a self-organizing dissipative system—an open biological system that maintains order through the continuous exchange of energy, information, and resources with its environment. This perspective highlights how aging in eusocial insects emerges not solely from individual decline, but from multilevel regulatory interactions linking cells, organisms, colonies, and ecosystems.

## Introduction

“The whole is greater than the sum of its parts,” wrote Aristotle in Metaphysics.

More than two millennia later, biology continues to rediscover the same truth: it is complexity, not simplicity, that sustains life. From cells assembling into tissues, to individuals forming colonies, to colonies creating ecosystems, the world shows that the persistence of life arises through integration—and through complexity itself.

The honeybee colony exemplifies the principle of complexity in an extraordinary way. It is a superorganism in which thousands of individuals coordinately function as a single being, and even the individual aging and death have been transformed into mechanisms of collective regulation. The plasticity of aging within the honeybee colony challenges the classical scientific view that senescence is both an inevitable and a stochastic product of damage accumulation. Instead, it reveals aging as a dynamic, emergent property of collective life.

In this Perspective, I present a conceptual framework that links honeybee aging with the broader logic of living systems—where physiological regulation, social organization, and environmental cycles converge to reveal aging as an emergent and reversible property of collective life.

### From individual decay to collective regulation

Classical life-history theory explains how natural selection shapes life-history strategies through the allocation of limited resources among three primary functions—growth, reproduction, and maintenance. Because resources are limited, investment in one function reduces investment in others, leading to evolutionary trade-offs that shape lifespan and senescence (Kirkwood and Kowald, 1997). Most evolutionary biologists therefore view aging as a non-adaptive and non-programmed process—a by-product of other biological and evolutionary mechanisms rather than a trait evolved for its own purpose. In this framework, senescence, defined as an age-associated decline in physiological function and survival (Kirkwood and Austad, 2000), arises as a residual cost of resource allocation and imperfect maintenance rather than as an adaptive mechanism under direct selection. One of the most widely recognized life-history trade-offs is the cost of reproduction, whereby increased investment in reproduction comes at the expense of somatic maintenance and survival, resulting in a negative relationship between reproductive effort and lifespan (Harshman and Zera, 2006).

A key mechanistic link between lifespan and reproduction is hormonal regulation, because hormones that promote reproduction—such as juvenile hormone (JH) in insects—often exert broad pleiotropic effects on metabolism, stress resilience, and somatic repair (Flatt et al., 2005). In most insects, elevated JH levels increase metabolic activity, reduce stress resistance, and suppress immune function, reinforcing the classical view that reproductive signaling comes at the expense of somatic maintenance (Herman and Tatar, 2001; Tu et al., 2002; Min and Tatar, 2006; Yamamoto et al., 2013).

Although the cost of reproduction is almost universal across the animal and plant kingdoms, it is reversed by eusocial insects, where reproductive individuals outlive their sterile colony members by orders of magnitude. Comparative analyses indicate that the evolution of eusociality has been associated with an approximately 100-fold increase in longevity of eusocial reproductive individuals relative to their solitary counterparts. Whereas adults of solitary insect species typically survive only weeks to a few months, queens and kings of eusocial insects may live for years or even decades, depending on the species (Keller and Genoud, 1997; Keller, 1998). In honeybees, queens may survive for up to five to 6 years (Winston, 1987). Such extreme longevity cannot be explained by genetic differences—since both reproductive and sterile castes share nearly identical genomes—nor by resource limitation alone. Instead, it points to a shift in the level of selection: from individuals competing for survival to colonies functioning as integrated systems (Münch et al., 2008; Münch and Amdam, 2010).

Honeybees provide a particularly striking example of this shift in the level of selection. They exhibit not only the extraordinary longevity of queens but also a remarkable plasticity of aging among workers (Münch and Amdam, 2010; Amdam, 2011). In workers, aging is manifested through coordinated physiological, endocrine, behavioral, and demographic changes rather than by chronological age alone (Amdam and Omholt, 2002; Rueppell et al., 2004). Consequently, the rate of worker aging can accelerate or decelerate depending on social and environmental context, thereby increasing colony adaptability to changing conditions. This plasticity is most apparent at the seasonal level, where worker lifespan ranges from only a few weeks in summer to several months in the long-lived winter phenotype (Winston, 1987). These seasonal changes are accompanied by profound physiological reorganization: summer workers are specialized for intensive foraging and rapid colony growth, whereas winter workers adopt a diutinus phenotype characterized by enhanced somatic maintenance, increased stress resistance, and prolonged lifespan (Amdam, 2011).

At the physiological core of this lifespan plasticity lies the modification of the juvenile hormone (JH)–vitellogenin (Vg) axis—an ancestral regulator of reproduction in oviparous animals (Münch et al., 2008; Münch and Amdam, 2010; Kodrík et al., 2023).

### Endocrine architecture of lifespan plasticity in honeybee workers: interplay between Vg and JH in the regulation of worker aging

In solitary species, JH and Vg are positively correlated: elevated JH stimulates Vg synthesis to promote oogenesis (Bownes, 1982; Kodrík et al., 2023). In honeybee workers, by contrast, Vg and JH are antagonistically linked—Vg downregulates JH production, and elevated JH in turn suppresses Vg expression (Münch and Amdam, 2010). As well documented in honeybees, Vg functions beyond its ancestral role as a yolk precursor, acting as an antioxidant and immune factor that promotes somatic maintenance and longevity (Kodrík et al., 2023). Resulting from the reciprocal negative feedback between JH and Vg—with JH being associated with a fast-aging physiological state and Vg with prolonged lifespan—the JH–Vg axis defines the physiological tempo of worker aging (Rutz et al., 1976; Pinto et al., 2000; Guidugli et al., 2005). High Vg and low JH characterize in-hive bees—typically younger workers in a slow-aging physiological state associated with enhanced somatic maintenance and extended lifespan. In contrast, high JH and low Vg define a fast-aging physiological state characteristic of older workers, particularly foragers, associated with elevated metabolic activity, reduced immune competence and antioxidant protection, and lower investment in somatic maintenance (Harrison and Fewell, 2002; Amdam et al., 2004; Seehuus et al., 2006; Chan et al., 2011). Moreover, cognitive aging, manifested as a decline in olfactory learning, has been shown to occur specifically in foragers, indicating that cognitive senescence is linked to behavioral state rather than chronological age alone (Behrends et al., 2007). Consequently, the transition between these states represents a physiological and demographic switch rather than a purely chronological one; the onset of foraging predicts mortality more accurately than age itself (Rueppell et al., 2007; Amdam, 2011).

The causal link between endocrine state and aging dynamics was confirmed by experimental manipulations. Elevating JH induced precocious foraging and early death, while enhancing Vg prolonged the in-hive phase and overall lifespan. Remarkably, these transitions are reversible: when colonies lose in-hive bees, foragers revert to nest tasks, restore Vg synthesis, suppress JH, and rejuvenate (Johnson, 2010). Reverted workers furthermore do not exhibit the age-associated cognitive deficits characteristic of normal foragers, indicating that behavioral and cognitive aspects of aging can also be reversed (Behrends et al., 2007). Aging, here, is not a one-way trajectory but a reversible physiological state—fluid, not fixed.

### Social pheromones as colony-wide regulators of aging

Individual endocrine states are synchronized through pheromonal communication. Three primer pheromones—queen mandibular pheromone (QMP), brood ester pheromone (BEP), and ethyl oleate (EO)—maintain colony homeostasis by linking social structure to the regulation of worker biological time. By modulating the balance between JH and Vg, these pheromonal signals influence behavioral maturation, reproductive physiology, and the rate of worker aging.

QMP suppresses JH and sustains high Vg levels, delaying behavioral maturation and maintaining workers in a slow-aging in-hive state. As the colony grows, the *per capita* QMP gradient in the colony weakens, which triggers—as an elegant demographic feedback loop—subsets of workers to elevate JH and initiate their foraging, resulting in their fast-tempo of aging (Johnson, 2010).

BEP reflects larval abundance and nutritional demand. When brood is scarce, BEP reduces JH and prolongs worker longevity; when brood is abundant, it increases JH and suppresses Vg, thereby promoting rapid behavioral maturation and transition to foraging (Pankiw et al., 1998; Le Conte et al., 2001; Johnson, 2010).

EO, produced by foragers, acts inversely to BEP: it delays the maturation of young workers, stabilizing the colony’s division of labor (Leoncini et al., 2004).

In summary, the three main primer pheromones provide complementary layers of social information that collectively regulate colony homeostasis and the pace of aging. Through these interconnected feedbacks, the pheromonal network aligns endocrine regulation with demographic and ecological context, ensuring coordinated senescence across the superorganism.

Seasonal environmental changes then impose a higher-order tempo of aging.

### Environmental entrainment of aging

**FIGURE 1:**
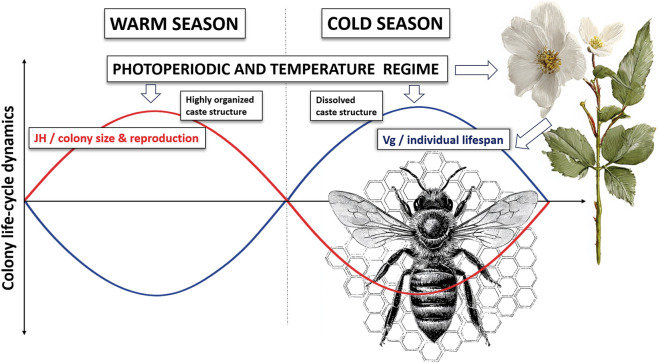
The JH–Vg axis as a regulator of honeybee colony dynamics and aging plasticity. Seasonal cycles of temperature and photoperiod shape the balance between juvenile hormone (JH) and vitellogenin (Vg), linking environmental conditions to reproduction, colony demography, and the tempo of worker aging. Through reciprocal feedback between JH and Vg, the colony transitions between alternative physiological attractor states: a summer expenditure state (high JH, low Vg, rapid worker turnover, colony expansion) and a winter maintenance state (high Vg, low JH, extended longevity, enhanced somatic maintenance). These dynamics are further modulated by plant phenology through brood-rearing intensity.

In temperate climates, temperature and photoperiod are the main regulators of the endocrine balance in bees, acting in both direct and indirect ways (Figure 1). Although honeybee colonies maintain brood temperature within a relatively narrow range through collective thermoregulation, ambient temperature remains an important seasonal cue, much like photoperiod. During the warm season, long days and high temperatures appear to directly elevate JH and suppress Vg in bees (Cherednikov, 1967; Fluri and Bogdanov, 1987; Huang and Robinson, 1995; Johnson, 2010; Koubová et al., 2021). In parallel to the direct influence, the temperature–photoperiodic regime shapes plant phenology (Fisogni et al., 2025). It results in the seasonal flow of nectar and pollen that stimulates brood-rearing intensity, which in turn feeds back on JH–Vg dynamics, mirroring in bees the effect of the temperature–photoperiodic regime itself (Di Pasquale et al., 2013; Corona et al., 2023). Collectively, both direct and indirect processes during warm season stimulate reproduction and colony expansion, but they also drive rapid worker turnover. Summer workers are short-lived, surviving only a few weeks (Winston, 1987).

As fall approaches, shorter days and lower temperatures reverse this pattern. Workers develop a resilient, long-lived diutinus phenotype, surviving for several months and characterized by low JH and high Vg levels, along with enhanced somatic maintenance and resource conservation (Huang and Robinson, 1995; Johnson, 2010; Koubová et al., 2021). In the diutinus state, the colony loses its typical division of labor; workers become generalists capable of performing multiple tasks as needed, ensuring colony survival under resource-limited conditions. The winter colony population is only a fraction of that in summer (Winston, 1987). Remarkably, both Vg and JH fluctuate in gradual seasonal patterns (Figure 1), apparently shifting with the progression of photoperiod and temperature throughout the year (Huang and Robinson, 1995; Johnson, 2010; Koubová et al., 2021).

The honeybee superorganism, through the Vg–JH axis, thus acts as a biological metronome, synchronizing its persistence with both ecosystem and planetary rhythms. From a systems perspective, this interplay illustrates how global environmental cycles are translated into physiological and cellular settings that, in turn, project back onto the tempo of individual aging. Thus, aging at the individual level not only emerges from the system—it becomes an adjustable dial: accelerating during expansion, slowing during conservation, and serving as a tool of collective adaptation rather than a symptom of decline.

Viewed from this perspective, the Vg–JH axis brings us back to the central question of life-history theory: how limited resources are allocated between growth, reproduction, and maintenance. The existence of alternative physiological states associated with different aging trajectories suggests that honeybee aging may be better understood not only through endocrine regulation but also through the organizational principles of dissipative systems, which maintain stability through dynamic transitions between contrasting modes of operation.

## Discussion

### The honeybee superorganism as a dual energetic system

According to r/K selection theory, species balance their investment between reproduction and survival depending on environmental stability (Pianka, 1970). While r-selected species are associated with unpredictable environments and maximization of their reproduction effort, K-selected species, which live under stable environment, invest heavily into longevity. Viewed through this lens, the summer honeybee colony functions in a mode of metabolic expansion that is oriented toward rapid growth and reproductive output, and resembling r-strategists. In winter, by contrast, the colony shifts to a K-like mode of metabolic retention—closed, conservative, and oriented toward physiological maintenance and survival. By oscillating between these two energetic states, the colony transcends the classical r/K framework: it acts as a “two-in-one strategist,” combining rapid turnover with long-term stability and demonstrating that endurance in nature depends not on stasis, but on balance between expansion and restraint.

Viewed from a systems perspective, this dynamic organization shares key characteristics with dissipative structures, which maintain stability through continuous flux, feedback regulation, and exchange with the environment.

### The honeybee colony as a dissipative system

Building on Prigogine’s Nobel-recognised theory of dissipative structures, biological systems persist only as open units—continuously exchanging energy, matter, and information with their environment. Life is sustained not by static equilibrium, which would correspond to stillness and death, but by organized disequilibrium: a state far from thermodynamic balance in which structure is actively maintained through continual metabolic flux (Prigogine and Nicolis, 1985; Capra, 2005).

Instead of settling into a single stable state, dissipative systems preserve order by oscillating or alternating between alternative regimes of activity. This dynamic behaviour is a widespread feature of biological organization, observed from metabolic and cell-cycle oscillations to circadian rhythms, seasonal physiology, and population cycles. It reflects the inherent nonlinearity of biological regulation, arising from cooperative molecular interactions and feedback networks that connect processes across levels of organization—from genes to hormones to ecosystems (Capra, 2005; Goldbeter, 2017; 2018).

The networks give rise to characteristic organizational patterns known as attractors: stable modes of cellular, organismal, or ecosystem organization to which the system returns after perturbation (Goldbeter, 2018). Transitions between attractors are controlled by feedback architecture and the state of key regulatory variables, such as hormone levels, nutrient availability, social signals, or environmental cues. Positive feedback between variables can stabilize distinct attractor states, observed as bistability, making them self-maintaining—as in the classical switch-like dynamics of REM-nonREM transitions during sleep (Lu et al., 2006), in the regulation of cyclin-dependent kinases during the cell cycle (Pomerening et al., 2003; Sha et al., 2003), or transitions between phases of mania and depression in bipolar disorders (Goldbeter, 2011). Negative feedback, by contrast, can generate self-sustained oscillations, exemplified by glycolytic oscillations, cell-cycle rhythms, and circadian clocks (Goldbeter, 2017). In many biological networks, these motifs interlock to produce stability not through constancy, but through coordinated temporal transitions (Goldbeter, 2018).

In the honeybee, the principles of dissipative organisation become visible at the scale of the colony. Although composed of thousands of individuals, the colony behaves as a single thermodynamic system whose stability depends on the continual circulation of energy, labour, and physiological states among its members. At the core of this regulatory architecture lies the JH–Vg axis, a mutually inhibitory endocrine–metabolic feedback loop. While this interaction is negative at the molecular level (Vg suppresses JH production and JH suppresses Vg expression), at the systems level it produces functional positive feedback: each physiological configuration reinforces itself while suppressing its alternative.

Consistent with the logic of dissipative structures, this architecture exhibits bistability, stabilising the colony in one of two alternative attractor states corresponding to the maintenance and expenditure modes described above. Within this framework, the JH–Vg axis thus functions as a biological decision-making module: it does not merely modulate endocrine or metabolic parameters, but selects between attractor states that define distinct life-history modes.

Seen in this light, ageing in honeybees is neither a fixed, irreversible trajectory nor a passive by-product of social organisation. Instead, it is a reversible state variable embedded in the colony’s dissipative dynamics—a dynamic modulation of biological time continually tuned to ecological conditions and collective demand.

## Future perspectives: from cells to ecosystems

Aristotle did not know words such as complexity or self-organization, yet he understood that nature is a hierarchically organized, multi-layered system in which each level arises from the one below but exceeds it in function and form. Honeybee aging exemplifies this principle: the way of aging within the colony is not confined to individual bees but emerges from the collective interplay of molecular, cellular and physiological processes with environment.

Yet, it would be a mistake to think that this is only about bees. The colony represents just one visible fragment of a far larger whole. The interaction between insect pollinators, of which the honeybee is only one, and flowering plants, a relationship that evolved some 130 million years ago, forms the foundation of most terrestrial ecosystems on Earth (Fisogni et al., 2025).

The endocrine mechanisms regulating honeybee life-history have been known for decades, yet only a systems-level perspective can reveal how these details fit together and offer a truer picture of life than the sum of its parts suggests.

## Data Availability

The original contributions presented in the study are included in the article/supplementary material, further inquiries can be directed to the corresponding author.
